# Peri‐ictal magnetic resonance imaging characteristics in dogs with suspected idiopathic epilepsy

**DOI:** 10.1111/jvim.16058

**Published:** 2021-02-09

**Authors:** Aran Nagendran, James Fraser McConnell, Luisa De Risio, Roberto José‐López, Rodrigo Gutierrez Quintana, Kelsey Robinson, Simon R. Platt, Daniel Sanchez Masian, Thomas Maddox, Rita Gonçalves

**Affiliations:** ^1^ Department of Veterinary Science, Small Animal Teaching Hospital University of Liverpool Cheshire United Kingdom; ^2^ Neurology/Neurosurgery Service, Centre for Small Animal Studies Animal Health Trust, Newmarket United Kingdom; ^3^ School of Veterinary Medicine University of Glasgow Glasgow United Kingdom; ^4^ Department of Small Animal Medicine and Surgery, College of Veterinary Medicine University of Georgia Athens Georgia USA

**Keywords:** canine, MRI, postictal, seizures

## Abstract

**Background:**

The pathophysiology of changes in magnetic resonance imaging (MRI) detected after a seizure is not fully understood.

**Objective:**

To characterize and describe seizure‐induced changes detected by MRI.

**Animals:**

Eighty‐one client‐owned dogs diagnosed with idiopathic epilepsy.

**Methods:**

Data collected retrospectively from medical records and included anatomical areas affected, T1‐, T2‐weighted and T2‐FLAIR (fluid‐attenuated inversion recovery) appearance, whether changes were unilateral or bilateral, symmetry, contrast enhancement, mass effect, and, gray and white matter distribution. Diffusion‐ and perfusion weighted maps were evaluated, if available.

**Results:**

Seizure‐induced changes were T2‐hyperintense with no suppression of signal on FLAIR. Lesions were T1‐isointense (55/81) or hypointense (26/81), local mass effect (23/81) and contrast enhancement (12/81). The majority of changes were bilateral (71/81) and symmetrical (69/71). The most common areas affected were the hippocampus (39/81) cingulate gyrus (33/81), hippocampus and piriform lobes (32/81). Distribution analysis suggested concurrence between cingulate gyrus and pulvinar thalamic nuclei, the cingulate gyrus and parahippocampal gyrus, hippocampus and piriform lobe, and, hippocampus and parahippocampal gyrus. Diffusion (DWI) characteristics were a mixed‐pattern of restricted, facilitated, and normal diffusion. Perfusion (PWI) showed either hypoperfusion (6/9) or hyperperfusion (3/9).

**Conclusions and Clinical Importance:**

More areas, than previously reported, have been identified that could incur seizure‐induced changes. Similar to human literature, DWI and PWI changes have been identified that could reflect the underlying metabolic and vascular changes.

**Abbreviations**
ADC: apparent diffusion coefficient
DWI: diffusion‐weighted imaging
FLAIR: fluid‐attenuated inversion recovery
MRI: magnetic resonance imaging
PWI: perfusion‐weighted imaging

## 1 INTRODUCTION

Magnetic resonance imaging (MRI) can identify seizure‐induced changes in the ictal and early postictal stages of seizure activity.^1^ The pathophysiology responsible for these changes is not fully understood. Seizure activity increases regional glucose and oxygen consumption and utilization. It has been proposed that to compensate for this increase in metabolic demand, compensatory regional hyperperfusion occurs.^2^ However, should sustained ictal activity persist, these compensatory mechanisms are no longer sufficient. This leads to hypoxia, lactic acidosis and failure of cellular homeostasis causing cellular swelling and increased membrane permeability. The cascade of metabolic changes subsequently leads to the formation of cytotoxic and vasogenic edema, a finding supported by experimentally induced seizures in rats.^3^ The formation of identifiable changes has been documented as time‐dependent^4^; especially dependent on duration of ictal activity.

Peri‐ictal changes are well‐documented in human literature. The prevailing conclusions from such investigations^1^ were that these changes occur in specific anatomical areas of the brain, they can be unilateral or bilateral, local or remote to the epileptogenic focus, and are partially or completely reversible; resulting in partial or complete resolution. Diffusion and perfusion‐weighted imaging support the presence of pathophysiological changes within the ictal tissue and suggest a time‐dependent evolution of these changes. The reversible nature and anatomical locations are described in 3 dogs with seizures of different etiologies,^5^ as well as the reversible nature of these changes.

The aims of our study were to further describe the changes and anatomical locations seen on MRI during the peri‐ictal stages in a group of dogs diagnosed with presumed idiopathic epilepsy and describe the anatomical locations affected. If available, we aimed to report if any changes were seen on diffusion‐ and perfusion‐weighted imaging.

## 2 MATERIALS AND METHODS

### 2.1 Study population and design

A multicenter retrospective cross‐sectional study was undertaken involving 4 centers; the Small Animal Teaching Hospital of the University of Liverpool, the Animal Health Trust, the Small Animal Hospital of the University of Glasgow and the Veterinary Teaching Hospital of the University of Georgia. The study protocol was approved by the Veterinary Research Ethics Committees of the participating centers; ref VREC724.

Medical records were searched for dogs presenting for investigation of epileptic seizures and that had peri‐ictal changes identified on MRI of the brain between 2010 and 2019. The terms “postictal” and “peri‐ictal” were searched within the imaging reports and the studies were identified. The inclusion criteria were further refined to include dogs that were diagnosed with suspected idiopathic epilepsy. The criteria included dogs that had an onset of seizures between 6 months and 6 years, had a normal cerebrospinal fluid (CSF) analysis, and did not develop progressive neurological deficits after 6 months after diagnosis. Cases were also included if repeated CSF analysis was normal, after an abnormal initial result and only if naive of medication that could influence CSF analysis. Further data collected included age, breed, sex, type and frequency of the most common seizure activity, and the duration of time between the last seizure and acquisition of MRIs. Exclusion criteria involved MRI abnormalities that were not consistent with reports in human and veterinary literature.

### 2.2 Magnetic resonance imaging

All the images were obtained from the following scanners: 1.5 T Philips Ingenia CX, Philips Healthcare, Best, Netherlands (University of Liverpool), 1.5T Signa Echospeed System, General Electric Medical System, Milwaukee (Animal Health Trust), 1.5T Siemens Magnetom Essenza, Siemens Healthcare, Germany (University of Glasgow), and, 3.0T Siemens Magnetom Skyra, Siemens Healthcare, Germany (University of Georgia). The dog positioning and anesthetic agents used were dependent on protocols of the individual centers enrolled. All the images were reviewed by a board‐certified radiologist (FMc). The imaging data obtained included anatomical areas affected, T1‐weighted, T2‐weighted and T2‐FLAIR (fluid‐attenuated inversion recovery) signal characteristics, whether the changes were unilateral or bilateral, symmetrical or asymmetrical, presence of contrast enhancement, mass effect and gray and white matter distribution. For those that had diffusion‐weighted imaging, the affected areas on conventional imaging were assessed. Diffusion weighted imaging (*B* = 0 and *B* = 800 or 1000) and apparent diffusion coefficient (ADC) maps were used for analysis. Similarly, for those that underwent perfusion‐weighted imaging, changes were assessed based on the affected areas on conventional MR imaging. Relative cerebral blood flow (rCBF), relative cerebral blood volume (rCBV) and mean transit time (MTT) maps were needed for interpretation. Quantitative values were obtained from the ADC, rCBF, rCBV, and MTT maps. They were obtained by manually drawing up to 3 regions of interest (ROIs) in each affected area identified on conventional imaging (T2‐FLAIR) and formulating a mean value. Care was taken to exclude large blood vessels from regions of interest.

Two separate control groups were also formulated: one to compare diffusion‐weighted changes and another to compare perfusion‐weighted changes. These control groups were composed of dogs diagnosed with primary myopathies or cranial nerve neuropathies without central nervous system involvement. Only images where lesions were free from artifacts were used for analysis. Quantitative ADC and perfusion map values (rCBF, rCBV and MTT) were obtained, from the respective control groups, of the areas that were known to be affected in the study groups. The median measurement values were subsequently obtained for each affected area based on each study group.

To assess whether the affected area had facilitated diffusivity, restricted diffusivity or normal diffusivity, the mean ADC values of the study group were compared to values obtained from manually drawn ROIs of the unaffected contralateral hemisphere (if unilateral changes) or compared to median values of the control group of dogs (if bilateral); that is, a higher comparative ADC value implied a facilitated diffusivity and a lower ADC value implied restrictive diffusivity. A similar method was applied to the mean perfusion measurements to assess whether an area showed evidence of hyperperfusion or hypoperfusion; that is, hyperperfusion was identified as increased rCBF, increased rCBV and reduced MTT, and hypoperfusion was identified as decreased rCBF, decreased rCBV, and prolonged MTT. Regions of interest in both diffusion and perfusion weighted imaging maps were defined as abnormal if the quantitative values were greater than 2 standard deviations of the control or contralateral hemisphere.

### 2.3 Statistical analysis

Coexisting or concurrent peri‐ictal changes were defined as identification of 2 or more lesions in different brain regions within the same animal. A concurrent lesion network was created using graphical network analytical methods, implemented in the igraph package available in R.^6^ A node referred to a brain location, with the size of the node being relative to the frequency of lesions at that location. An edge (link) referred to a coexisting lesion at a different location, with edge weight being relative to frequency of coexisting lesions. This undirected connected graph (network) consisting of 10 nodes and 35 edges was then examined for group structure via an optimal community structure algorithm, with the aim of defining groups (communities) of commonly seen concurrent lesions. Briefly, this algorithm partitions the network structure to maximize its modularity function (*Q*) and identify the optimal community configuration consisting of groups with many more internal connections than expected at random.^7^ A Fisher's Exact test was performed to assess whether there was significant association between the diffusion and perfusion‐weighted findings and was performed on a standard statistical software package (SPSS: Statistical Package for the Social Sciences V.22.0.1, SPSS). This was therefore only applied to the cases which had both diffusion and perfusion measurements documented. The measurements assessed were qualitative values; restricted and facilitated diffusivity, and, hypoperfusion and hyperperfusion. A *p*‐value of .05 was deemed significant.

## 3 RESULTS

### 3.1 Animals

A total of 81 dogs fulfilled the inclusion criteria. The study group included the following breeds; Border Collie (9), Staffordshire Bull Terrier (9), German Shepherd (6), Boxer (5), Mixed Breed (5) Border Terrier (4), Labrador (4), French Bulldog (3), Pug (3), Springer Spaniel (3), Cavalier King Charles Spaniel (3)Hungarian Vizsla (2), Miniature Schnauzer (2), Lurcher (2), and one each of the following breeds Australian Shepherd dog, Beagle, Bichon Frise, Chihuahua, Cocker Spaniel, Doberman, Elkhound, English Bulldog, German Shorthaired Pointer, Griffon Bruxellois, Golden Retriever, Irish Setter, Jack Russell Terrier, Lakeland Terrier, Petite Basset Griffon Vendeen, Rottweiler, Shih tzu, Siberian Husky, Spanish Water Dog, West Highland White Terrier, and Wirehaired Fox Terrier. There were 54 males (36 were neutered) and 27 females (15 were neutered).

Unrelated comorbidities documented were thrombocytopenia (2) hypothyroidism (1), hyperadrenocorticism, and cranial cruciate ligament rupture (1). The median age of onset of seizures was 30 months (interquartile range 16‐45.5).

At the time of presentation, 43/81 (53%) cases had evidence of seizure activity within 24 hours prior to imaging acquisition, and 64/81 (79%) had evidence of seizure within a 48‐hours prior to imaging. The longest duration recorded between last seizure and imaging acquisition was 7 days (n = 3). 70/81 (86%) cases presented with generalized seizures, 8/81 had both generalized and focal seizures, and 3/81 had partial signs only. The seizure incidence identified in the cases was as follows; 43/81 (53%) presented with cluster seizures, 23/81 (28%) presented with isolated seizures, and 15/81 (19%) presented in status epilepticus.

### 3.2 Magnetic resonance imaging

**FIGURE 1:**
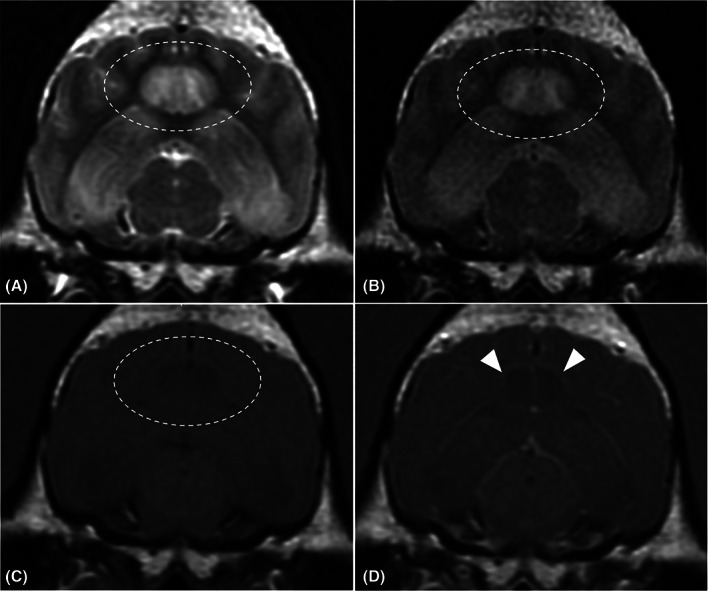
Magnetic Resonance transverse images of 1 case at the level of the mesencephalon; A, T2‐weighted, B, T2‐weighted fluid‐attenuated inversion recovery (FLAIR), C, T1‐weighted precontrast and D, T1‐weighted postcontrast. These images highlight T1‐hypointense, T2‐hyperintense areas affecting the cingulate gyrus (encircled area) and the hippocampus. These hyperintensities do not suppress on FLAIR. Contrast enhancement is identified in the periphery of the affected areas including the meninges (white arrows) and the ependymal border

**FIGURE 2:**
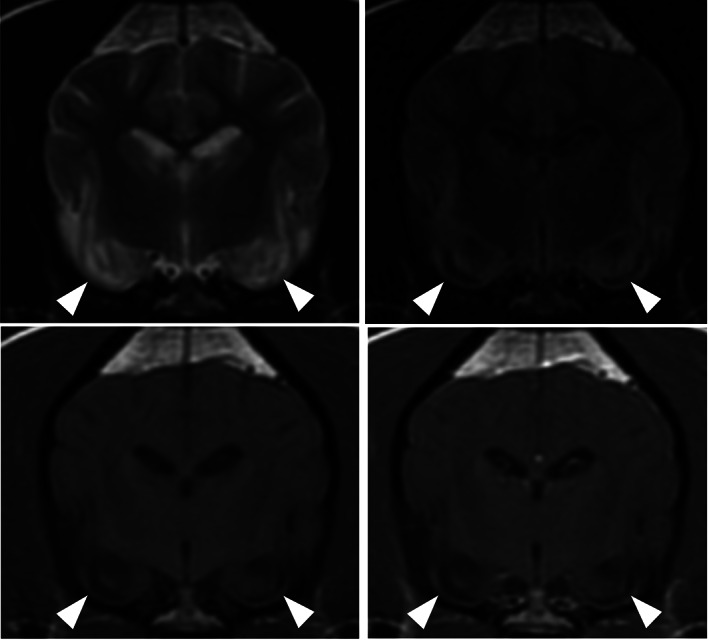
Magnetic Resonance transverse images at the level of the piriform lobes; T2‐weighted (A), T2‐weighted fluid‐attenuated inversion recovery (FLAIR) (B0, T1‐weighted precontrast (C), and T1‐weighted postcontrast (D). These images highlight a T1‐hypointense, T2‐hyperintense cavitated areas affecting the amygdala and piriform lobes (white arrowheads). These areas do suppress on FLAIR and have mild rim contrast enhancement; suggestive of areas of necrosis

**FIGURE 3:**
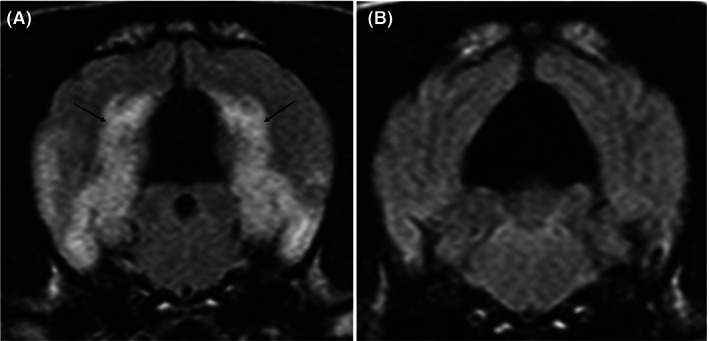
Magnetic resonance imaging of the same case at initial presentation (A), 1 week apart (B). Both images are T2‐FLAIR. Identifying a near complete resolution of T2‐weighted hyperintensities occupying the temporal and occipital cortex

On conventional MRI, all the affected areas (n = 81) were T2‐hyperintense with no signal suppression on FLAIR. On T1‐weighted imaging, 55/81 had T1‐isointense and 26/81 had T1‐hypointense areas. Contrast enhancement was identified in 12/81 dogs with 8/12 having meningeal enhancement around the affected areas and 4/12 parenchymal enhancement. Local mild mass effect (Figure 1) was identified in 23/81 of the cases but there was no evidence of herniation as a result of the changes identified in any dog. Bilateral lesions were seen in 71/81 cases with 69/71 having a symmetrical appearance. Both bilateral areas and an additional unilateral area were identified in 3/81 cases, and 6/81 had unilateral changes only. Two dogs had evidence of presumed malacic changes to the piriform lobes bilaterally (Figure 2). These affected areas were characterized by T1‐hypointense, T2‐hyperintense cavitary lesions which did not uptake any contrast; both these cases were Staffordshire bull terriers. Repeat imaging was obtained in 3/81 cases; in 2 dogs this was performed 1 week after the initial MRI and in 1 dog after 10 months. Full resolution of changes was identified in the latter dog and in one of the early scans (Figure 3). In the other MRI performed 1 week after initial investigations, there was a marked reduction in the size of the original changes. All 3 of the repeated scans had abnormal initial CSF analysis, namely mild increase in total nucleated cell concentration and protein concentration (Supporting Information File S1). On repeated analysis, all 3 cases had normal CSF analysis.

**FIGURE 4:**
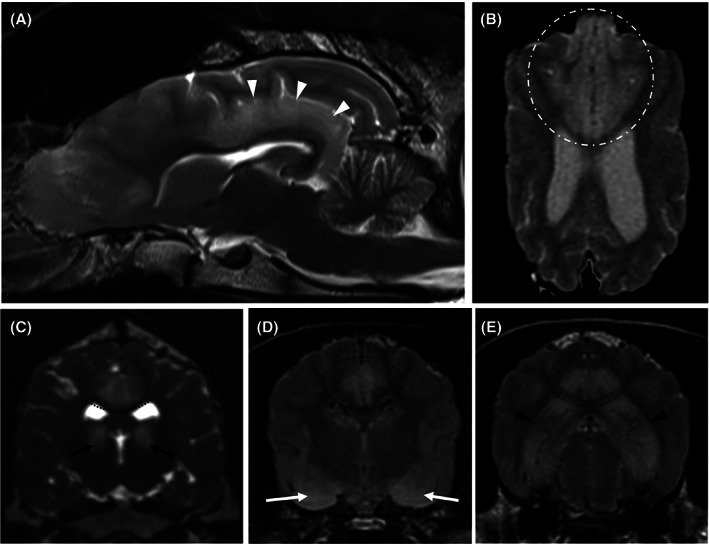
Magnetic resonance images highlighting the distribution of seizure‐induced changes; A, T2‐weighted sagittal image showing T2‐hyperintense cingulate gyrus extending into the parahippocampal gyrus (white arrows), B, T2‐weighted dorsal image showing homogeneous bilateral symmetrical T2‐hyperintensity affecting the gray matter of the frontal and olfactory lobes (dotted and hashed white encircled area), C, T2‐weighted transverse image showing well‐defined wedge‐shaped bilateral symmetrical T2‐hyperintensities affecting the region of the pulvinar thalamic nuclei (black arrows), D, T2‐FLAIR transverse image showing bilateral T2‐hyperintense piriform lobes, and, E, T2‐FLAIR transverse image showing bilateral symmetrical homogenous T2‐hyperintensities affecting the hippocampus. Images C‐E also show bilateral symmetrical T2‐hyperintensities affecting the cingulate gyri

Lesions were identified in 10 anatomical areas of the brain in this study: hippocampus, cingulate gyrus, piriform lobe (including amygdala), occipital lobe, frontal lobe, pulvinar thalamic nucleus, caudate nucleus, temporo‐parietal lobes and olfactory lobes (Figure 4). The prevalence of these lesions identified the hippocampus (n = 39), cingulate gyrus (n = 33) and the piriform lobes (n = 32) being the most commonly affected sites.

**FIGURE 5:**
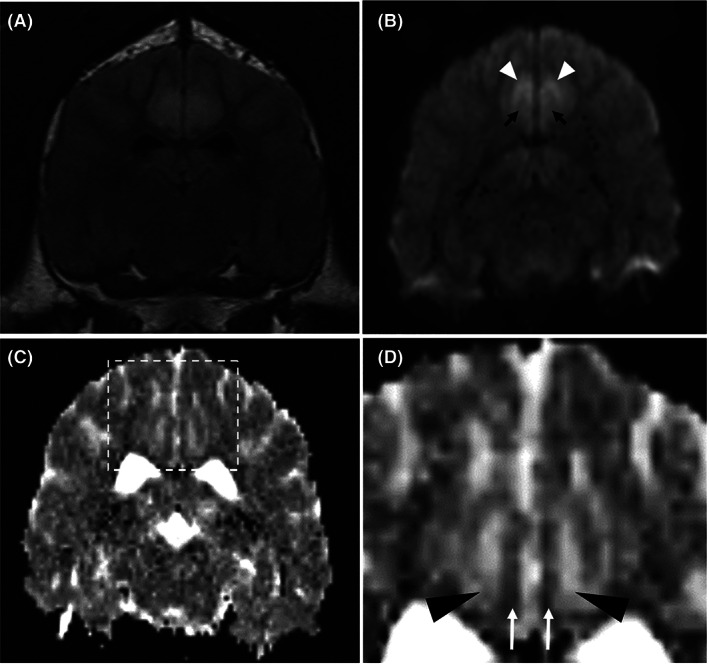
Diffusion‐weighted magnetic resonance imaging (DWI) of 1 case showing a transverse T2‐weighted fluid‐attenuation inversion recovery (FLAIR) image (A), a transverse DWI b = 1000 image (B) and a transverse apparent diffusion coefficient (ADC) (C and D); at the level of the caudal diencephalon. The T2‐hyperintense areas appear to have very high signal intensity affecting the gray matter (white arrowhead) and mildly higher signal intensity affecting the white matter (black arrows) on the DWI image. The mildly increased signal affecting the white matter could be due to T2‐shine through phenomenon. On the ADC map, there is high signal within the white matter (black arrowhead) and low signal affecting the gray matter (white arrow). This could indicate facilitated diffusion in the white matter and restrictive diffusion in the gray matter of the cingulate gyri

Diffusion weighted imaging (Figure 5) was obtained from 21/81 cases and the control group was composed of 40 cases. The control group consisted of dogs diagnosed with idiopathic vestibular neuropathy (17/40), idiopathic facial nerve neuropathy (14/40), idiopathic vestibular and facial nerve neuropathy (4/40), masticatory muscle myositis (2/40), otitis media (2/40), and idiopathic trigeminal neuropathy (1/40). The mean values were obtained from the piriform lobes, hippocampi, parahippocampal gyri, occipital lobes, cingulate gyri, olfactory lobes, frontal lobes, and pulvinar nuclei. Only 19 cases were available for analysis due to susceptibility artifact presence over the affected areas in 2 cases (see Supporting Information File S2). There were 34 bilateral affected regions and 4 unilateral affected regions that were assessed. Of the bilateral affected regions, all regions had the same type of diffusivity when compared to the contralateral affected region. The median variance from the contralateral hemisphere was 0.06 × 10^−3^ mm^2^/s (range 0‐0.21 × 10^−3^ mm^2^/s). On the ADC map, 19/38 regions had evidence of facilitated diffusivity, 14/38 regions had evidence of restricted diffusivity, 4/38 regions had normal diffusivity and one region had a mixture of restrictive and facilitative diffusion; cingulate gyrus gray matter was restrictive and cingulate gyrus white matter was facilitative (Figure 4). There were 36 regions localized to the gray matter and 3 regions localized to the white matter. In the gray matter affected areas, there was a mixture of diffusivity: 18 had facilitative diffusivity, 14 had restrictive diffusivity and 4 had normal diffusivity. All 3 white matter affected regions had facilitated diffusivity and were all identified in the cingulate gyrus. Evaluating by case, 4/19 cases had facilitative patterning in all affected regions, 4/19 had restrictive patterning in all affected regions and 2/19 had normal diffusivity patterning in all affected regions. The rest of the cases (9/19) had a mixture of diffusivity affecting each region. When assessing affected anatomical areas, the pulvinar thalamic nuclei (affected bilaterally in 3 cases) and amygdala (affected bilaterally in 3 cases) had only one type of diffusivity documented whereas all anatomical areas had evidence of facilitative diffusivity.

Perfusion weighted imaging was obtained from 9/81 cases from 1 of the 4 centers and 20 cases from the same center composed the control group used for analysis. The relative maps were obtained through dynamic susceptibility contrast MRI. Scanning measurements can be found in Supporting Information File S3. The median values for the control group were obtained for the left and right piriform lobe, left and right hippocampus, left and right frontal gray matter, left and right occipital gray matter and the left and right cingulate gyrus (all areas affected within the 9 cases in the study group) (see Supporting Information File S4). There was evidence of hyperperfusion in 3/9 cases with increased rCBV, increased rCBF and reduced MTT. There was evidence of hypoperfusion in 6/9 cases with decreased rCBV, decreased rCBF and prolonged MTT. Both diffusion and perfusion weighted imaging were available in 9/81 cases; there was no significant association (*P* = .34) between the diffusion findings and the perfusion findings identified.

### 3.3 Distribution data

**FIGURE 6:**
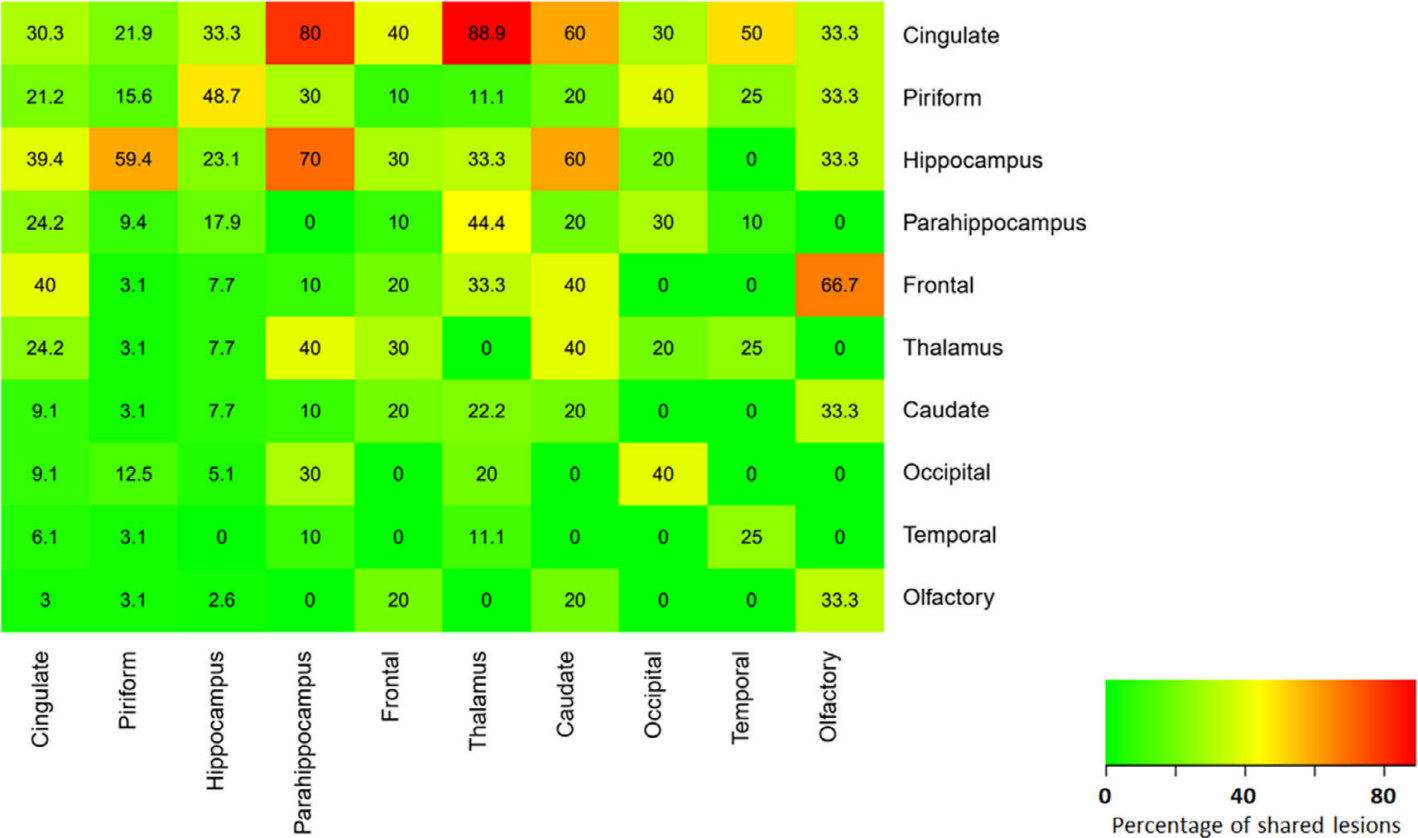
Heat map showing the proportion of coexisting lesions at each location, with each expressed as a percentage of total lesions at that location. The x‐axis represents the independent location, whereas the y‐axis represents the concurrent dependent location. For example, if a cingulate lesion was present, 30.3% were cingulate‐only lesions, but 21.9% of piriform, 33.3% of hippocampal and 80% of parahippocampal lesions were present concurrently

**FIGURE 7:**
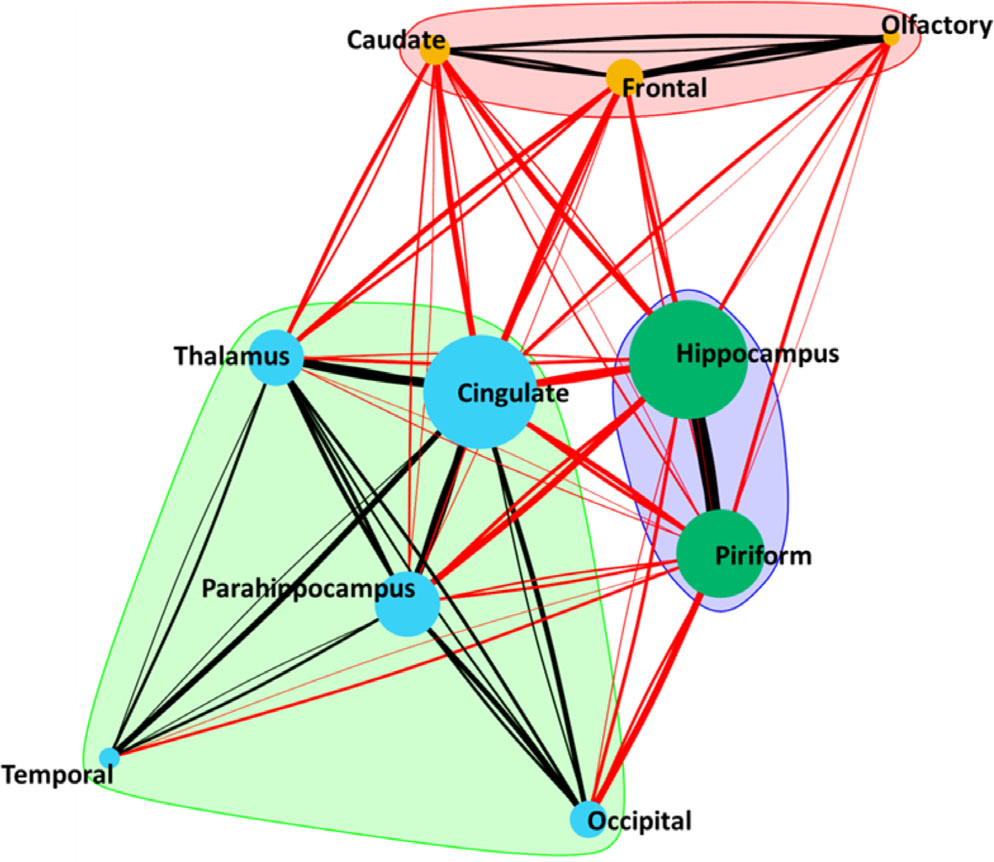
Concurrent lesion network showing coexisting lesions and identified groupings of commonly concurrent lesions. Cingulate lesions were those most frequently seen in conjunction with lesions at other locations and are hence placed at the center of the network. Node diameter is proportion to the frequency of lesions identified at that location and the edges (connecting lines) are proportional to the frequency of coexisting lesions. Black edges show coexisting lesion within a group (intragroup) and red edges show coexisting lesions between groups (intergroup)

Distribution analysis was carried out to identify if there was an association between certain anatomical areas. The results reveal that multiple affected locations were more often found than just a single location (Figure 6). This revealed the following relationships: 80% and 89% of the all cases of cingulum lesions observed were present when a parahippocampal lesion and pulvinar thalamic lesion was identified respectively, and 70% of all cases of hippocampal lesions observed were seen in the presence of a parahippocampal lesion. Connectivity of certain locations was seen (Figure 7) and imply that there is increased connectivity between olfactory lobe, caudate nuclei and frontal, between the piriform lobe and the hippocampus, and between the cingulate gyrus, parahippocampal gyrus, occipital lobe, pulvinar thalamic nuclei, and the temporal lobe.

## 4 DISCUSSION

The most common acute peri‐ictal MR changes include bilateral symmetrical T2‐ and T2‐FLAIR hyperintense, T1‐hypointense area with mild contrast uptake and have tendency for local mild mass effect. The most common sites affected included the hippocampus, cingulate gyrus, and piriform lobes; although other locations have now been identified. Abnormalities in the diffusion and perfusion maps have also been recognized in this study; which might reflect the underlying pathophysiological processes.

The imaging abnormalities identified on conventional MRI in this study, are similar to those reported elsewhere.^5^ Studies in rodents have identified that the T2‐weighted hyperintense areas are a result of ictal‐related edema or early or developing gliosis.^1^, ^8^ The patterning of peri‐ictal gadolinium enhancement seen correlated well with what has been previously reported in human and animal studies; with a discrete area of leptomeningeal enhancement around the affected T2‐hyperintense areas most commonly identified.^5^, ^9^ The proposed mechanism for this is that prolonged ictal activity leads to local lactic acidosis, an increase in carbon dioxide and neurotransmitter release, leading to an increase in local vascular permeability.^9^

There are currently no studies in humans or in domestic species correlating seizure‐induced changes with neuropathological findings. One hypothesis behind the increased T2 signal could be related to swelling or hypertrophy of glial cells leading to prolonged T2‐relaxation times. This is based on theories on dysregulation of aquaporin‐4 channels^10^ and failure of Na/K pumps^11^ leading to the formation of vasogenic and cytotoxic edema.^3^

Conventional imaging cannot distinguish between cytotoxic and vasogenic edema, thus more advanced techniques such as diffusion‐weighted imaging have been used. Diffusion weighted imaging assesses the movement and distribution of water molecules within a given time within a specific location. Changes in signal on DWI and on ADC maps in dogs with kainite‐induced status epilepticus are caused by ictal activity.^12^ A reduction in ADC corresponds with decreased diffusivity which could be directly associated with cytotoxic intracellular edema due to intracellular ictal‐driven excitotoxic mechanisms. Other theories for a decrease in ADC values include macrophagic, microglial, and astrocytic proliferation.^3^ An increase in ADC values is commonly associated with vasogenic edema formation or progressive neuronal loss. Studies in humans have targeted their efforts to evaluate the fluctuations in ADC values as they are thought to reflect neuronal metabolic dynamics associated with seizure activity.^13^, ^14^ Some of these studies identify a reduction in ADC values; while others have identified a transient increase in ADC values within the peri‐ictal and interictal period.^3^, ^12^, ^13^, ^14^, ^15^ The variability of findings could be time‐dependent and follow certain stages; stage 1 initial regional hyperperfusion with no detectable ADC change, stage 2: high ADC values due to vasogenic edema formation, stage 3: reduction in ADC values resulting from cytotoxic edema formation, and, stage 4: increased ADC values due to progressive neuronal loss.^16^ In idiopathic epileptic dogs, ADC values have been shown to be increased in the amygdala within the interictal period^17^ and it was speculated that this change could be secondary to neuronal cell loss or gliosis.

The results of this study similarly identified a variety of patterns of diffusivity. The small size of the group limits statistical power. However, the variation might suggest a similar dynamic time‐dependent change to the ictal and paraictal tissues. In some cases, both high and low signal were identified on the ADC maps, suggesting that these stages might overlap. Consistent changes in the piriform lobes and the amygdala are similar to interictal findings in idiopathic epileptic dogs.^17^ A subgroup of our cases had a normal signal on ADC maps but an increased signal on DWI. This could be associated with different underlying mechanisms including that DWI is not only dependent on ADC map but also on the T2‐weighted appearance. Therefore, having a high signal on DWI and a normal signal on ADC could imply “T2‐shine through”. Other possibilities for a normal signal on an ADC map are that there could be combined effects of vasogenic and cytotoxic edema, or, cytotoxic edema and gliosis, affecting the same area of tissue. Lastly, it is possible that the images acquired in the 3‐Tesla scanner, compared to the 1.5‐Tesla, gave a better resolution of images, making it easier to differentiate between areas of reduced and increased signal. It is well established in humans that there is an initial local hyperperfusion during ictal activity followed by hypoperfusion in the postictal period.^18^ Our study group has identified that these changes in perfusion occur in dogs.

The affected anatomical areas identified are more extensive than those previously reported.^5^ The cingulate gyrus in particular features as one of the most prevalent areas affected, alongside the piriform lobe and the hippocampus. Another new location identified in this study was the medial pulvinar thalamic nucleus. It was interesting to note that all the thalamic affected areas were in this particular location. Electrophysiologic and resting state functional connectivity human studies demonstrate that the medial pulvinar nucleus is particularly important in genesis and reciprocal propagation of seizure activity with the mesial temporal structures.^19^, ^20^

This study identified that some affected areas had potential influence over the likelihood of other areas being affected. One possibility is that two locations could have a contiguous relationship. Examples of this association include the cingulate gyrus and parahippocampal gyrus and the parahippocampal gyrus and hippocampus, which have been identified in our study as frequently affected at the same time. Another suggestion is that there likely are projection fibers connecting certain other areas with each other. An example of this would the pulvinar thalamic nuclei and certain areas of the cortex due to thalamocortical relay circuits. The anatomical areas identified, and the connectivity proposed, resembles an already defined circuitry; the Papez's circuit. The Papez's circuit is a fundamental component of the limbic system and therefore a control of emotional expression and memory. The structures include the entorhinal cortex, hippocampus, fornix, mammillary bodies, parahippocampal gyrus, cingulate gyrus, amygdala, and anterior thalamus. Abnormalities of Papez's circuitry has been identified in human patients with mesial temporal epilepsy.^21^

CSF abnormalities were identified in 3 cases, which had normalization of measurements on repeat analysis within a few days without specific treatment. Increased protein and total nucleated cell concentration are a transient phenomena in humans and suspected in dogs although this finding remains controversial.^22^, ^23^, ^24^

As part of the inclusion of this study, we identified two cases that had bilateral necrotic changes to the amygdala and piriform lobes. These changes have been documented in experimental studies as well as other retrospective imaging‐based studies.^12^, ^25^ However, these have only been described in the interictal period of idiopathic epileptic dogs. In addition to these studies, hippocampal atrophy has also been observed in dogs.^26^, ^27^, ^28^ Such peri‐ictal changes might be the prequel to such irreversible changes; brain atrophy and cortical laminar necrosis, as a result of sustained epileptic discharges in humans.^29^

There are several limitations in this study, mostly related to its retrospective nature. There were few repeated studies (only performed in 3 cases) and for this reason it was difficult to completely exclude other differentials for seizure activity such as metabolic, vascular, or neoplastic changes. Also, without this information, pertinent data related to evidence of persistent changes and whether they were related to the areas of suspected cytotoxic edema could not be evaluated. The perfusion maps were based on T2* based sequences and some of the DWI were also gradient echo‐based. This results in susceptibility artifacts around air‐filled chambers such as the frontal sinuses and tympanic bullae. In some cases, the artifacts distorted the areas of interest making it difficult to analyze and interpret. Lastly, it was difficult to fully define the boundaries between piriform cortex and amygdala on conventional imaging; due to close proximity of the structures and similarities in appearance in T1‐ and T2‐weighted sequences. Volumetry using higher resolution MRI and 3‐dimensional software would need to be incorporated to analyze amygdaloid changes in more detail.^30^

The variability of the MRI changes in this study implies limited clinical utility. These abnormalities can be a mixture of local and remote changes and thus adjunctive diagnostic utility of electroencephalography should be used to aid the diagnosis of the epileptogenic focus.^31^

Ischemic infarcts can be difficult to differentiate from the MRI changes described in this study. However, the combination of hyperperfusion, increased T2‐signal and restricted diffusion is uncharacteristic of ischemia.^32^ Also, ictal changes will not align vascular territories.^33^

## CONFLICT OF INTEREST DECLARATION

Authors declare no conflict of interest.

## OFF‐LABEL ANTIMICROBIAL DECLARATION

Authors declare no off‐label use of antimicrobials.

## INSTITUTIONAL ANIMAL CARE AND USE COMMITTEE (IACUC) OR OTHER APPROVAL DECLARATION

Authors declare no IACUC or other approval was needed.

## HUMAN ETHICS APPROVAL DECLARATION

Authors declare human ethics approval was not needed for this study.

## Supporting information


**Data S1** File 1.

**Data S2** File 2: Quantitative values based on ADC maps generated and interpretation of cases. Unilateral locations were compared to the contralateral hemisphere. Bilateral locations were compared to the control group

**Data S3** File 3: The contrast administered was Gadovist (Bayer). A concentration of 1 mmol/mL was given at a dose rate of 0.1 mmol/kg with a saline flush; using a dual head pressure injector. A flow rate of 3‐5 mL/s was administered; dependent on patient size. A dynamic echo planar imaging (EPI) sequence was used 50 dynamic scans acquired. Contrast injection was started after 10 dynamic acquisitions were obtained. Each dynamic scan of the whole brain took less than 2 seconds.

**Data S4** File 4: Perfusion values based on perfusion maps and interpretation of cases. Unilateral locations were compared to the contralateral hemisphere. Bilateral locations were compared to the median value of the control group.
