# Supplementary material for: Peri‐ictal magnetic resonance imaging characteristics in dogs with suspected idiopathic epilepsy
Source: J Vet Intern Med. 2021 Feb 9;35(2):1008–17. doi: 10.1111/jvim.16058 (PMC7995424; doi:10.1111/jvim.16058)
Supplement: Supplementary file 1 — Data S1 File 1. [file JVIM-35-1008-s001.pdf]

Supplementary File 1

	Initial Investigation		Time after intial investigation (days)	Repeat Investigation	
	TNCC [cells/uL]	Protein [g/L]		TNCC [cells/ul]	Protein [g/L]
**Case 1**	12	0.45	7	2	0.13
**Case 2**	8	0.39	7	0	0.1
**Case 3**	10	0.43	304	0	0

All cases had cerebellomedullary cisternal taps. The reference ranges used for TNCC was <5 cells/uL and protein was < 0.35g/L.

TNCC: Total nucleated cell concentration
