# Supplementary material for: Peri‐ictal magnetic resonance imaging characteristics in dogs with suspected idiopathic epilepsy
Source: J Vet Intern Med. 2021 Feb 9;35(2):1008–17. doi: 10.1111/jvim.16058 (PMC7995424; doi:10.1111/jvim.16058)
Supplement: Supplementary file 2 — Data S2 File 2: Quantitative values based on ADC maps generated and interpretation of cases. Unilateral locations were compared to the contralateral hemisphere. Bilateral locations were compared to the control group [file JVIM-35-1008-s002.pdf]

## Supplementary File 2

*Quantitatuve values based on ADC maps generated and interpretation of cases. Unilateral locations were compared to the contralateral hemisphere. Bilateral locations were compared to the control group.*

| Case | Location | Bilateral [B] / Unilateral [U] | GM VALUES | | | | | | | | WM VALUES | | | | | Mean control values | | SD Control ADC values | | Variance from control | | Overall Findings | |
|---|---|---|---|---|---|---|---|---|---|---|---|---|---|---|---|---|---|---|---|---|---|---|---|
| | | | B1000 | B800 | GM ADC | Hemisphere side | GM Value: 10^-3 mm²/s Max | Min | Mean | SD | WM ADC | WM Value: 10^-3mm²/s Max | Min | Mean | SD | GM | WM | GM | WM | GM | WM | GM | WM |
| Case 1 | Piriform | B | - | High | High | R | 1.27 | 0.67 | 0.97 | 0.2 | - | - | - | - | - | 0.86 | - | 0.05 | - | 0.11 | - | Facilitated | - |
| | | | - | High | High | L | 1.23 | 0.66 | 0.95 | 0.21 | - | - | - | - | - | 0.85 | - | 0.04 | - | 0.1 | - | Facilitated | - |
| | Hippocampus | B | - | High | High | R | 1.31 | 0.91 | 1.11 | 0.12 | - | - | - | - | - | 0.74 | - | 0.06 | - | 0.37 | - | Facilitated | - |
| | | | - | High | High | L | 1.1 | 0.93 | 1.01 | 0.05 | - | - | - | - | - | 0.75 | - | 0.08 | - | 0.26 | - | Facilitated | - |
| Case 2 | Piriform | B | - | High | Low | R | 0.78 | 0.51 | 0.65 | 0.15 | - | - | - | - | - | 0.86 | - | 0.05 | - | -0.21 | - | Restricted | - |
| | | | - | High | Low | L | 0.79 | 0.52 | 0.66 | 0.08 | - | - | - | - | - | 0.85 | - | 0.04 | - | -0.19 | - | Restricted | - |
| | Amygdala | B | - | High | High | R | 1.13 | 1.10 | 1.08 | 0.06 | - | - | - | - | - | 0.86 | - | 0.08 | - | 0.22 | - | Facilitated | - |
| | | | - | High | High | L | 1.37 | 1.23 | 1.29 | 0.05 | - | - | - | - | - | 0.85 | - | 0.1 | - | 0.44 | - | Facilitated | - |
| | Hippocampus | B | - | High | - | R | 0.93 | 0.78 | 0.86 | 0.05 | - | - | - | - | - | 0.74 | - | 0.06 | - | 0.12 | - | Facilitated | - |
| | | | - | High | - | L | 1.09 | 0.75 | 0.92 | 0.12 | - | - | - | - | - | 0.75 | - | 0.08 | - | 0.17 | - | Facilitated | - |
| | Parahippocampal gyrus | B | - | High | - | R | 1.12 | 0.93 | 1.01 | 0.05 | - | - | - | - | - | 0.78 | - | 0.12 | - | 0.23 | - | Facilitated | - |
| | | | - | High | - | L | 1.2 | 0.83 | 0.97 | 0.1 | - | - | - | - | - | 0.78 | - | 0.1 | - | 0.19 | - | Facilitated | - |
| | Occipital | B | - | High | - | R | 1.13 | 0.73 | 0.93 | 0.13 | - | - | - | - | - | 0.8 | - | 0.04 | - | 0.13 | - | Facilitated | - |
| | | | - | High | - | L | 1.5 | 0.89 | 1.11 | 0.19 | - | - | - | - | - | 0.81 | - | 0.04 | - | 0.3 | - | Facilitated | - |
| Case 3 | Cingulate gyrus | B | - | High | High | R | 1.08 | 0.84 | 0.96 | 0.07 | - | - | - | - | - | 0.79 | - | 0.08 | - | 0.17 | - | Facilitated | - |
| | | | - | High | - | L | 1.03 | 0.86 | 0.95 | 0.07 | - | - | - | - | - | 0.80 | - | 0.07 | - | 0.15 | - | Facilitated | - |
| | Frontal | B | - | High | - | R | 0.89 | 0.75 | 0.81 | 0.05 | - | - | - | - | - | 0.69 | - | 0.06 | - | 0.12 | - | Facilitated | - |
| | | | - | High | - | L | 1.1 | 0.74 | 0.84 | 0.08 | - | - | - | - | - | 0.69 | - | 0.06 | - | 0.15 | - | Facilitated | - |
| Case 4 | Piriform | B | High | - | Low | R | 0.83 | 0.61 | 0.72 | 0.04 | - | - | - | - | - | 0.86 | - | 0.05 | - | -0.14 | - | Restricted | - |
| | | | - | - | - | L | 0.86 | 0.6 | 0.73 | 0.05 | - | - | - | - | - | 0.85 | - | 0.04 | - | -0.12 | - | Restricted | - |
| | Amygdala | B | - | - | - | R | 1.15 | 0.88 | 1.02 | 0.02 | - | - | - | - | - | 0.86 | - | 0.08 | - | 0.16 | - | Facilitated | - |
| | | | - | - | - | L | 1.18 | 0.93 | 1.06 | 0.03 | - | - | - | - | - | 0.85 | - | 0.1 | - | 0.21 | - | Facilitated | - |
| | Occipital | B | - | - | - | R | 1.12 | 0.77 | 0.98 | 0.09 | - | - | - | - | - | 0.8 | - | 0.04 | - | 0.18 | - | Facilitated | - |
| | | | - | | - | L | 1.08 | 0.75 | 0.92 | 0.05 | - | - | - | - | - | 0.81 | - | 0.04 | - | 0.11 | - | Facilitated | - |
| Case 5 | Piriform | U | High | - | Normal | R | 0.88 | 0.69 | 0.78 | 0.04 | - | - | - | - | - | 0.86 | - | 0.05 | - | -0.08 | - | Restricted | - |
| | Hippocampus | B | High | - | Normal | R | 1.38 | 0.28 | 0.7 | 0.32 | - | - | - | - | - | 0.74 | - | 0.06 | - | -0.04 | - | Restricted | - |
| | | | High | - | Normal | L | 0.82 | 0.52 | 0.67 | 0.23 | - | - | - | - | - | 0.75 | - | 0.08 | - | -0.08 | - | Restricted | - |
| Case 6 | Piriform | B | - | High | Low | R | 0.96 | 0.55 | 0.76 | 0.05 | - | - | - | - | - | 0.86 | - | 0.05 | - | -0.1 | - | Restricted | - |
| | | | - | High | Low | L | 0.84 | 0.48 | 0.66 | 0.07 | - | - | - | - | - | 0.85 | - | 0.04 | - | -0.19 | - | Restricted | - |
| Case 7 | Cingulate gyrus | B | - | High | - | R | - | - | - | - | High | 1.31 | 0.91 | 1.1 | 0.12 | - | 0.82 | - | 0.11 | - | 0.28 | - | Facilitated |
| | | | - | High | - | L | - | - | - | - | High | 1.39 | 0.87 | 1.07 | 0.16 | - | 0.83 | - | 0.1 | - | 0.24 | - | Facilitated |
| Case 8 | Olfactory | B | - | High | High | R | 0.97 | 0.86 | 0.91 | 1.08 | - | - | - | - | - | 0.68 | - | 0.04 | - | 0.23 | - | Facilitated | - |
| | | | - | High | - | L | 1.08 | 0.88 | 1.01 | 0.08 | - | - | - | - | - | 0.70 | - | 0.04 | - | 0.31 | - | Facilitated | - |
| Case 9 | Occipital | U | - | High | High | L | 0.88 | 0.52 | 0.7 | 0.08 | - | - | - | - | - | 0.81 | - | 0.04 | - | -0.11 | - | Restricted | - |
| Case 10 | Cingulate gyrus | B | High | - | Low | R | 0.74 | 0.54 | 0.64 | 0.15 | High | 1.61 | 0.92 | 1.21 | 0.19 | 0.79 | 0.7 | 0.08 | 0.11 | -0.15 | 0.51 | Restricted | Facilitated |
| | | | High | - | - | L | 0.73 | 0.4 | 0.57 | 0.15 | High | 1.62 | 0.96 | 1.39 | 0.16 | 0.8 | 0.71 | 0.07 | 0.1 | -0.23 | 0.68 | Restricted | Facilitated |
| | Pulvinar | B | High | - | High | R | 1.46 | 0.87 | 1.16 | 0.15 | - | - | - | - | - | 0.75 | - | 0.16 | - | 0.41 | - | faciltated | - |
| | | | High | - | - | L | 1.45 | 0.65 | 1.06 | 0.19 | - | - | - | - | - | 0.76 | - | 0.12 | - | 0.3 | - | Facilitated | - |
| | Hippocampus | B | High | - | Low | R | 1.15 | 0.55 | 0.76 | 0.21 | - | - | - | - | - | 0.74 | - | 0.06 | - | 0.02 | - | Normal | - |
| | | | High | - | - | L | 1.01 | 0.61 | 0.81 | 0.2 | - | - | - | - | - | 0.75 | - | 0.08 | - | 0.06 | - | Normal | - |
| | Occipital | B | High | - | Low | R | 0.82 | 0.42 | 0.7 | 0.14 | - | - | - | - | - | 0.8 | - | 0.04 | - | -0.1 | - | Restricted | - |
| | | | High | - | - | L | 0.95 | 0.56 | 0.72 | 0.15 | - | - | - | - | - | 0.81 | - | 0.04 | - | -0.09 | - | Restricted | - |
| Case 11 | Piriform | B | High | - | High | R | 1.32 | 0.7 | 0.97 | 0.1 | - | - | - | - | - | 0.86 | - | 0.05 | - | 0.11 | - | Facilitated | - |
| | | | High | - | - | L | 1.24 | 0.64 | 0.94 | 0.1 | - | - | - | - | - | 0.85 | - | 0.04 | - | 0.09 | - | Facilitated | - |
| Case 12 | Cingulate Gyrus | B | High | - | - | R | 0.79 | 0.45 | 0.62 | 0.2 | - | - | - | - | - | 0.79 | - | 0.08 | - | -0.17 | - | Restricted | - |
| | | | High | - | - | L | 0.77 | 0.41 | 0.59 | 0.14 | - | - | - | - | - | 0.8 | - | 0.07 | - | -0.21 | - | Restricted | - |
| | Hippocampus (Head) | B | - | - | - | R | 1.22 | 0.7 | 0.95 | 0.15 | - | - | - | - | - | 0.74 | - | 0.06 | - | 0.21 | - | faciltated | - |
| | | | - | - | - | L | 1.26 | 0.76 | 0.96 | 0.15 | - | - | - | - | - | 0.75 | - | 0.08 | - | 0.21 | - | Facilitated | - |
| Case 13 | Pulvinar | B | High | - | High | R | 1.71 | 0.53 | 1.05 | 0.23 | - | - | - | - | - | 0.75 | - | 0.16 | - | 0.3 | - | Facilitated | - |
| | | | High | - | High | L | 1.57 | 0.71 | 1.14 | 0.26 | - | - | - | - | - | 0.76 | - | 0.12 | - | 0.38 | - | Facilitated | - |
| | Cingulate gyrus | B | High | - | Low | R | 0.86 | 0.49 | 0.62 | 0.09 | - | - | - | - | - | 0.79 | - | 0.08 | - | -0.17 | - | Restricted | - |
| | | | High | - | Low | L | 0.75 | 0.45 | 0.59 | 0.08 | - | - | - | - | - | 0.80 | - | 0.07 | - | -0.21 | - | Restricted | - |
| | Occipital | B | High | - | High | R | 1.28 | 0.66 | 1.07 | 0.17 | - | - | - | - | - | 0.8 | - | 0.04 | - | 0.27 | - | Facilitated | - |
| | | | - | - | - | L | 1.52 | 0.63 | 0.98 | 0.3 | - | - | - | - | - | 0.81 | - | 0.04 | - | 0.17 | - | Facilitated | - |
| Case 14 | Piriform | B | - | - | - | R | 0.89 | 0.82 | 0.86 | 0.2 | - | - | - | - | - | 0.86 | - | 0.05 | - | 0 | - | Normal | - |
| | | | - | - | - | L | 0.9 | 0.79 | 0.84 | 0.21 | - | - | - | - | - | 0.85 | - | 0.04 | - | -0.01 | - | Normal | - |
| Case 15 | Frontal | B | - | - | Low | R | 0.78 | 0.54 | 0.66 | 0.2 | - | - | - | - | - | 0.69 | - | 0.06 | - | -0.03 | - | Normal | - |
| | | | - | - | - | L | 0.76 | 0.49 | 0.63 | 0.18 | - | - | - | - | - | 0.69 | - | 0.06 | - | -0.06 | - | Normal | - |
| Case 16 | Piriform | B | - | - | Low | R | 0.79 | 0.55 | 0.67 | 0.05 | - | - | - | - | - | 0.86 | - | 0.05 | - | -0.19 | - | Restricted | - |
| | | | - | - | - | L | 0.78 | 0.58 | 0.68 | 0.06 | - | - | - | - | - | 0.85 | - | 0.04 | - | -0.17 | - | Restricted | - |
| | Left cingulate gyrus | U | - | - | - | L | - | - | - | - | High | 1.38 | 1.03 | 1.19 | 0.12 | - | 0.8 | - | 0.1 | - | 0.39 | - | Facilitated |
| | Occipital | B | - | - | Low | R | 0.79 | 0.59 | 0.69 | 0.04 | - | - | - | - | - | 0.8 | - | 0.04 | - | -0.11 | - | Restricted | - |
| | | | - | - | Low | L | 0.78 | 0.57 | 0.68 | 0.03 | - | - | - | - | - | 0.81 | - | 0.04 | - | -0.13 | - | Restricted | - |
| Case 17 | Piriform | B | - | - | Low | R | 0.86 | 0.62 | 0.74 | 0.04 | - | - | - | - | - | 0.86 | - | 0.05 | - | -0.12 | - | Restricted | - |
| | | | - | - | - | L | 0.88 | 0.64 | 0.76 | 0.04 | - | - | - | - | - | 0.85 | - | 0.04 | - | -0.09 | - | Restricted | - |
| | Amygdala | B | - | High | High | R | 1.41 | 0.78 | 1.1 | 0.12 | - | - | - | - | - | 0.86 | - | 0.08 | - | 0.24 | - | Facilitated | - |
| | | | - | - | - | L | 1.35 | 0.82 | 1.09 | 0.09 | - | - | - | - | - | 0.86 | - | 0.1 | - | 0.23 | - | Facilitated | - |
| Case 18 | Frontal | U | - | High | Low | R | 0.66 | 0.42 | 0.54 | 0.08 | - | - | - | - | - | 0.69 | - | 0.06 | - | -0.15 | - | Restricted | - |
| Case 19 | Piriform lobe | B | High | - | High | R | 0.96 | 0.69 | 0.83 | 0.06 | - | - | - | - | - | 0.86 | - | 0.05 | - | -0.03 | - | Normal | - |
| | | | - | - | High | L | 1.1 | 0.77 | 0.86 | 0.08 | - | - | - | - | - | 0.85 | - | 0.04 | - | 0.01 | - | Normal | - |
| | Hippocampus | B | - | - | Low | R | 0.7 | 0.44 | 0.57 | 0.07 | - | - | - | - | - | 0.74 | - | 0.06 | - | -0.17 | - | Restricted | - |
| | | | - | - | Low | L | 0.67 | 0.42 | 0.55 | 0.08 | - | - | - | - | - | 0.75 | - | 0.08 | - | -0.2 | - | Restricted | - |

*GM- gray matter, WM- white matter, ADC- apparent diffusion coefficient*
