# Supplementary material for: Peri‐ictal magnetic resonance imaging characteristics in dogs with suspected idiopathic epilepsy
Source: J Vet Intern Med. 2021 Feb 9;35(2):1008–17. doi: 10.1111/jvim.16058 (PMC7995424; doi:10.1111/jvim.16058)
Supplement: Supplementary file 3 — Data S3 File 3: The contrast administered was Gadovist (Bayer). A concentration of 1 mmol/mL was given at a dose rate of 0.1 mmol/kg with a saline flush; using a dual head pressure injector. A flow rate of 3‐5 mL/s was administered; dependent on patient size. A dynamic echo planar imaging (EPI) sequence was used 50 dynamic scans acquired. Contrast injection was started after 10 dynamic acquisitions were obtained. Each dynamic scan of the whole brain took less than 2 seconds. [file JVIM-35-1008-s004.pdf]

Supplementary File 3

The contrast administered was Gadovist (Bayer). A concentration of 1mmol/ml was given at a dose rate of 0.1mmol/kg with a saline flush; using a dual head pressure injector. A flow rate of 3-5ml/s was administered; dependant on patient size. A dynamic echo planar imaging (EPI) sequence was used 50 dynamic scans acquired. Contrast injection was started after 10 dynamic acquisitions were obtained. Each dynamic scan of the whole brain took less than 2 seconds.
