# Supplementary material for: Peri‐ictal magnetic resonance imaging characteristics in dogs with suspected idiopathic epilepsy
Source: J Vet Intern Med. 2021 Feb 9;35(2):1008–17. doi: 10.1111/jvim.16058 (PMC7995424; doi:10.1111/jvim.16058)
Supplement: Supplementary file 4 — Data S4 File 4: Perfusion values based on perfusion maps and interpretation of cases. Unilateral locations were compared to the contralateral hemisphere. Bilateral locations were compared to the median value of the control group. [file JVIM-35-1008-s003.pdf]

Supplementary File 4

Perfusion values based on perfusion maps and interpretation of cases. Unilateral locations were compared to the contralateral hemisphere. Bilateral locations were compared to the median value of the control group.

Case	Affected location		Affected ROI*				Comparative Location	Median Control Population/ Contralateral hemisphere ROI*				Interpretation
	Right	Left	CBF	CBV	MTT	TTP		CBF	CBV	MTT	TTP	
1	Piriform Lobe		3.21	125.34	39.09	38.28	Control	1.62	57.23	31.76	32.21	Hyperperfusion
		Piriform Lobe	3.14	124.59	39.62	38.19	Control	1.71	58.34	31.77	32.21	Hyperperfusion
	Hippocampus		2.67	111.89	41.91	40.28	Control	2.27	101.88	34.55	31.99	Hyperperfusion
		Hippocampus	3.2	133.6	41.72	40.15	Control	2.25	72.90	33.59	32.56	Hyperperfusion
2	Occipital Lobe		1.53	66.16	43.28	42.19	Control	3.08	136.83	34.10	33.40	Hypoperfusion
		Occipital lobe	1.25	53.68	42.96	41.18	Control	4.35	155.99	32.02	30.62	Hypoperfusion
3	Cingulate Gyrus		1.83	75.48	41.34	40.48	Control	5.22	179.37	32.15	31.16	Hypoperfusion
		Cingulate Gyrus	1.51	62.39	41.39	40.9	Control	4.64	147.58	32.26	31.80	Hypoperfusion
4	Piriform Lobe		1.13	55.75	46.2	35.87	Control	1.62	57.23	31.76	32.21	Hypoperfusion
		Piriform Lobe	1.11	28.7	45.96	45.94	Control	1.71	58.34	31.77	32.21	Hypoperfusion
5	Piriform Lobe		5.01	209.75	41.86	41.86	Left Piriform	2.35	103.18	43.96	43.96	Hyperperfusion
6	Piriform Lobe		1.6	53.21	39.57	39.98	Control	1.62	57.23	31.76	32.21	Hypoperfusion
		Piriform Lobe	1.02	30.22	49.63	48.83	Control	1.71	58.34	31.77	32.21	Hypoperfusion
7	Hippocampus		1.11	51.91	46.66	49.14	Control	2.27	101.88	34.55	31.99	Hypoperfusion
		Hippocampus	1.63	70.83	43.47	41.62	Control	2.25	72.90	33.59	32.56	Hypoperfusion
	Cingulate Gyrus		1.92	81.07	42.14	41.41	Control	5.22	179.37	32.15	31.16	Hypoperfusion
		Cingulate Gyrus	1.25	52.66	42.13	41.08	Control	4.64	147.58	32.26	31.80	Hypoperfusion
8	Frontal Lobe		7.3	201.68	27.62	27.21	Control	4.49	151.44	32.02	30.62	Hyperperfusion
		Frontal lobe	5.58	150.99	27.04	27.03	Control	4.66	150.06	32.53	31.70	Hyperperfusion
9		Occipital Lobe	6.03	195	32.33	31.54	Right Occipital	5.20	133.06	32.12	32.21	Hyperperfusion

CBF: cerebral blood flow, CBV: cerebral blood volume, MTT: mean transit time, ROI: region of interest.
